# Patterns of lung aeration assessed through electrical impedance tomography in paediatric patients undergoing elective surgery: insights from a prospective and observational data-registry

**DOI:** 10.1186/s44158-025-00254-x

**Published:** 2025-06-23

**Authors:** Daniela Rosalba, Grazia Meneghetti, Federico Verdina, Chiara Solai, Danila Azzolina, Laura Petronio, Matteo Guaraglia, Raffaella Buscaglia, Giulio Saviolo, Gaia Furlan, Filippo Vietti, Daniele Biasucci, Savino Spadaro, Rachele Simonte, Edoardo De Robertis, Federico Longhini, Serena Penpa, Michele Ubertazzi, Elena Panuccio, Paolo Aluffi, Stefano De Cillà, Matteo Brucoli, Rosanna Vaschetto, Gianmaria Cammarota

**Affiliations:** 1 Department of Traslational Medicine, Università del Piemonte Orientale, Novara, Italy; 2 Anaesthesia and Intensive Care, Azienda Ospedaliero Universitaria “Maggiore Della Carità”, Novara, Italy; 3 Department of Ambiental Science and Prevention, Università Degli Studi Di Ferrara, Ferrara, Italy; 4 Anaesthesia and Intensive Care, Ospedale Sant’Andrea, Vercelli, Italy; 5 Anaesthesia and General Intensive Care, Azienda Ospedaliero Universitaria Di Alessandria, Alessandria, Italy; 6 Department of Clinical Science and Translational Medicine, Tor Vergata’ University of Rome, Rome, Italy; 7 Department of Traslational Medicine, Unversità Degli Studi Di Ferrara, Ferrara, Italy; 8 Department of Medicine and Surgery, University of Perugia, Perugia, Italy; 9 Department of Medical and Surgical Sciences, Magna Graecia University, Catanzaro, Italy; 10 Research and Innovation Department (DAIRI), Azienda Ospedaliero Universitaria Di Alessandria, Alessandria, Italy; 11 Pediatric Surgery, Azienda Ospedaliero Universitaria “Maggiore Della Carità”, Novara, Italy; 12 Pediatric Ortho-Trauma Surgery, Azienda Ospedaliero Universitaria “Maggiore Della Carità”, Novara, Italy; 13 ENT Division, Azienda Ospedaliero Universitaria “Maggiore Della Carità”, Novara, Italy; 14 Eye Clinic, Azienda Ospedaliero Universitaria “Maggiore Della Carità”, Novara, Italy; 15 Maxillofacial Surgery, Azienda Ospedaliero Universitaria “Maggiore Della Carità”, Novara, Italy

**Keywords:** Electrical impedance tomography, Spontaneous breathing, Mechanical ventilation, Pediatrics, Monitoring

## Abstract

**Background:**

The impact of anaesthesia on lung function during paediatric surgery remains an area of active investigation. Understanding respiratory mechanics under different anaesthetic approaches is crucial for optimising pulmonary management in this vulnerable population.

**Objective:**

To assess ventilation distribution changes during different phases of anaesthesia in paediatric patients, using electrical impedance tomography (EIT).

**Methods:**

This observational study included 76 paediatric surgical patients—57 under controlled mechanical ventilation (CMV) and 19 breathing spontaneously. EIT assessed lung ventilation at multiple timepoints (T1-T6), analyzing regional distribution (ROIs) and center of ventilation (CoV).

**Results:**

In the CMV group, ventilation progressively shifted toward ventral lung regions (*p* < 0.0001 from T1 to T2, T3, T4, T5) with a contemporaneously reduced ventilation switching from T1 to T2 (*p* = 0.005), T3 (*p* < 0.0001), T4 (*p* = 0.001), and T5 (*p* < 0.0001). Ventilation normalised upon restoration of spontaneous breathing at the end of surgery. In the same group, CoV shifted toward non-dependent lung regions from T1 to T2, T3, T4, and T5 (*p* < 0.0001) and returned to baseline at T6. Overall, no modifications were observed in the spontaneous breathing group.

**Conclusions:**

In paediatric surgical patients, contrariwise to spontaneous breath where no modifications occurred, CMV induced a progressive redistribution of ventilation towards the ventral lung regions, at the expense of the dorsal zones. These changes were reversible with the recovery of spontaneous breathing.

**Trial registration:**

NCT06370507.

**Supplementary Information:**

The online version contains supplementary material available at 10.1186/s44158-025-00254-x.

## Background

Anaesthesia is closely associated with the development of atelectasis and hypoventilation, conditions that may persist in the postoperative period, thereby increasing the risk of pulmonary complications [1]. The primary cause of this phenomenon is the loss of muscle tone, particularly in charge of the diaphragm, which becomes less effective in maintaining differential pressures between the abdominal and thoracic cavities [2]. This results in an increased intrapleural pressure, especially in the dependent regions of the lung, compressing alveoli and small airways and leading to their collapse, with subsequent atelectasis and redistribution of ventilation [3, 4]. These effects are more pronounced in paediatric patients, who exhibit a higher thoracic wall compliance and significantly lower functional residual capacity compared to adults, making them more susceptible to pulmonary derecruitment during anaesthesia [5]. Moreover, paediatric patients undergo significant changes in the respiratory system from birth to adolescence, including lung and alveolar growth, ossification of the thoracic cage, and increased muscle tone. These developmental differences explain the impossibility of generalising physiological parameters and research findings across different paediatric age groups [6].

In this context, electrical impedance tomography (EIT) emerges as a valuable tool enabling real-time and non-invasive monitoring of ventilation distribution [7]. EIT is a functional imaging technique suitable for monitoring both adult and paediatric patients [8]. Its functioning is based on the assessment of tissue electrical properties, particularly bioimpedance, as low-intensity currents are applied through the chest via electrodes positioned at the fourth to sixth intercostal spaces [7]. This technology enables the reconstruction of detailed ventilation maps and the calculation of clinically relevant derived parameters through dedicated software [9]. In adult patients, EIT is used in the operating room to monitor pulmonary ventilation distribution during anaesthesia, mechanical ventilation, and surgical procedures (such as pneumoperitoneum) that may impair ventilation patterns [10]. This approach helps to optimise ventilatory settings, minimising atelectasis and derecruitment [11]. Despite its potential, research on paediatric patients remains limited, primarily concentrating on specific age groups or clinical contexts [12, 13]. Remarkably, the behaviour of the lung during paediatric and neonatal anaesthesia has been the subject of few investigations so far [12, 13].

The present study aims to address the current gap of knowledge in EIT application to evaluate ventilation distribution during the different phases of anaesthesia in paediatric surgical procedures. The goal is to provide insights to improve clinical practice and research in managing ventilation for paediatric patients undergoing anaesthesia for surgery.

## Methods

Data for the present study were retrieved from a prospective and observational data registry focused on EIT application during surgical procedures conducted in the operating room of the paediatric unit at Azienda Ospedaliero Universitaria “Maggiore della Carità” Novara, Italy. Following the approval of the hospital’s Ethics Committee and registration on Clinical Trial (NCT06370507 in April 2024), screening commenced. Once informed consent was obtained from both the parents of the paediatric patient in accordance with local regulations, the enrolment was performed.

### Patients

Inclusion criteria were the following: age ≤ 14 years (I), ASA classification I–III (II), no history of pulmonary disease (III), undergoing elective or non-elective non-thoracic surgery (IV), treated with either general anaesthesia or sedation (V). Exclusion criteria were identified as follows: patients aged > 14 years (I), ASA classification IV–V (II), history of known pulmonary disease (III), history of respiratory infections or fever within the 2 weeks preceding surgery (IV), history of laryngospasm or bronchospasm in the 3 weeks preceding surgery (V), lack of parental informed consent (VI), regional anaesthesia alone (VII), occurrence of severe anaesthetic complications during the procedure (VIII), and problems in the recording or interpretation of EIT registrations (IX).

### Study design

Patients received anaesthesia per standard operating room practice and institutional protocol, based on the type of surgery and clinical condition. The anaesthetic approaches included: (1) controlled mechanical ventilation (CMV) in deep sedation with laryngeal mask or under general anaesthesia with endotracheal tube and (2) spontaneous breathing in sedation eventually combined to regional anaesthesia. In the first group, patients underwent deep sedation or general anaesthesia induced with Propofol 2–4 mg/kg, opioid (Fentanyl 1–2 mcg/kg or Remifentanil 0.25–0.8 mcg/kg/min for 3 min), and neuromuscular blocking agents (Rocuronium 0.6 mg/kg at induction) in the majority of cases requiring endotracheal intubation. Deep sedation and general anaesthesia were maintained using intravenous agents (1% Propofol in Target Control Infusion mode with a target concentration of 5–7 mcg/ml at the effect site) or halogenated gases (Sevoflurane 2–3%), in combination with Remifentanil at 0.125–0.25 mcg/kg/min.

In the second group, patients were administered 1% Propofol using Target Control Infusion mode, with a target concentration of 3–5 mcg/ml at the effect site, and an opioid (Fentanyl 1–2 mcg/kg) to achieve sedation while maintaining spontaneous breathing or providing brief mask-assisted ventilation only immediately after induction. Regional anaesthesia was combined with sedation for some of these patients.

In CMV group, tidal volume was set in the range of 6–8 ml/kg. In both groups, end-tidal carbon dioxide was maintained between 30 and 35 mmHg when monitored. As this was an observational study, no standardised protocol was followed for ventilatory parameter adjustments during anaesthesia; modifications were made according to clinical need as assessed by the attending anaesthesiologist.

Data of interest were collected at six distinct time points. Each registration lasted approximately 1 min and included at least six consecutive breaths. The recorded phases were as follows: (T1) pre-induction, with the patient awake and breathing spontaneously, (T2) induction, with assisted ventilation via facial mask, (T3) spontaneous breathing or mechanical ventilation at 5 min post-induction, (T4) spontaneous breathing or mechanical ventilation at 30 min post-induction, (T5) late phase, just before awakening, and (T6) recovery, with spontaneous breathing resumed and removal of any airway devices used.

### EIT

In the operating room, after venous access placement, standard continuous monitoring of vital signs was initiated. All recruited patients were monitored using EIT via a chest belt equipped with 16 electrodes, positioned between the fourth and sixth intercostal spaces. The belt size was adjusted based on patient weight and height. Noteworthy, the EIT belt was never moved or removed from the patient, and its position was checked before every single recording to assure the recording quality. Data acquisition was performed through the PulmoVista 500 device (Dräger Medical GmbH), which recorded intra-tidal changes in mean thoracic impedance for each breath. This device measures impedance variations primarily related to air flow, generating real-time two-dimensional images of lung ventilation. These images were divided into four regions of interest (ROIs), namely: ventral, mid-ventral, mid-dorsal, and dorsal lung regions. The derived EIT data were obtained through a dedicated software (Dräger Medical GmbH).

### Data acquisition

For each patient, an anonymised identifier was provided. When available, demographic and clinical data—including age, gender, body weight, height, body mass index (BMI), American Society of Anesthesiologists (ASA) risk classification, and type of surgical procedure—were reported. Vital parameters (heart rate, non-invasive blood pressure, peripheral oxygen saturation, bispectral index) and ventilatory parameters (respiratory rate, inspiratory fraction of oxygen, end-tidal carbon-dioxide) were acquired when available. For patients in CMV, both set and generated parameters were recorded when acquired, including inspiratory pressure, positive end-expiratory pressure, mean airway pressure, and airway plateau pressure.

### Endpoints

The study aimed to describe the distribution of ventilation across the ROIs during the different phases of anaesthesia and assessing ventilation allocation using the center of ventilation (CoV) (primary endpoint). CoV was expressed as a percentage of the dorsoventral chest diameter, where a value of 50% represented an equal distribution of ventilation between ventral and dorsal lung regions. Higher values reflected a ventral shift, while lower values indicated a dorsal distribution. Also, any association between the patterns of ventilation distribution and the anaesthesia plan was investigated (secondary endpoint).

### Statistical analysis

As the data for the present investigation were obtained from a prospective observational data-registry and given the exploratory nature of the study, no sample size calculation was carried out. Data were synthetised by median and 25th–75th percentile for continuous variables and absolute and relative frequency (%) for categorical variables. Assessment of time effects on EIT variables, oxygenation and respiratory mechanics parameters, as well as hemodynamics and sedation depth were analysed using repeated measures ANOVA. Post-hoc tests were performed through Bonferroni correction method. A multilevel mixed-effects linear regression model (MMEM) for prediction of ventilation distribution according to bispectral index (BIS) was estimated accounting for repeated measurements by including a random intercept term defined by the patient’s identification number. The models obtained were also adjusted for patient characteristics, specifically age and BMI.

The model estimates, along with standard errors, 95% confidence intervals, and *p*-values were reported. A *p*-value of less than 0.05 was considered to identify a statistically significant effect.

## Results

**Fig. 1:**
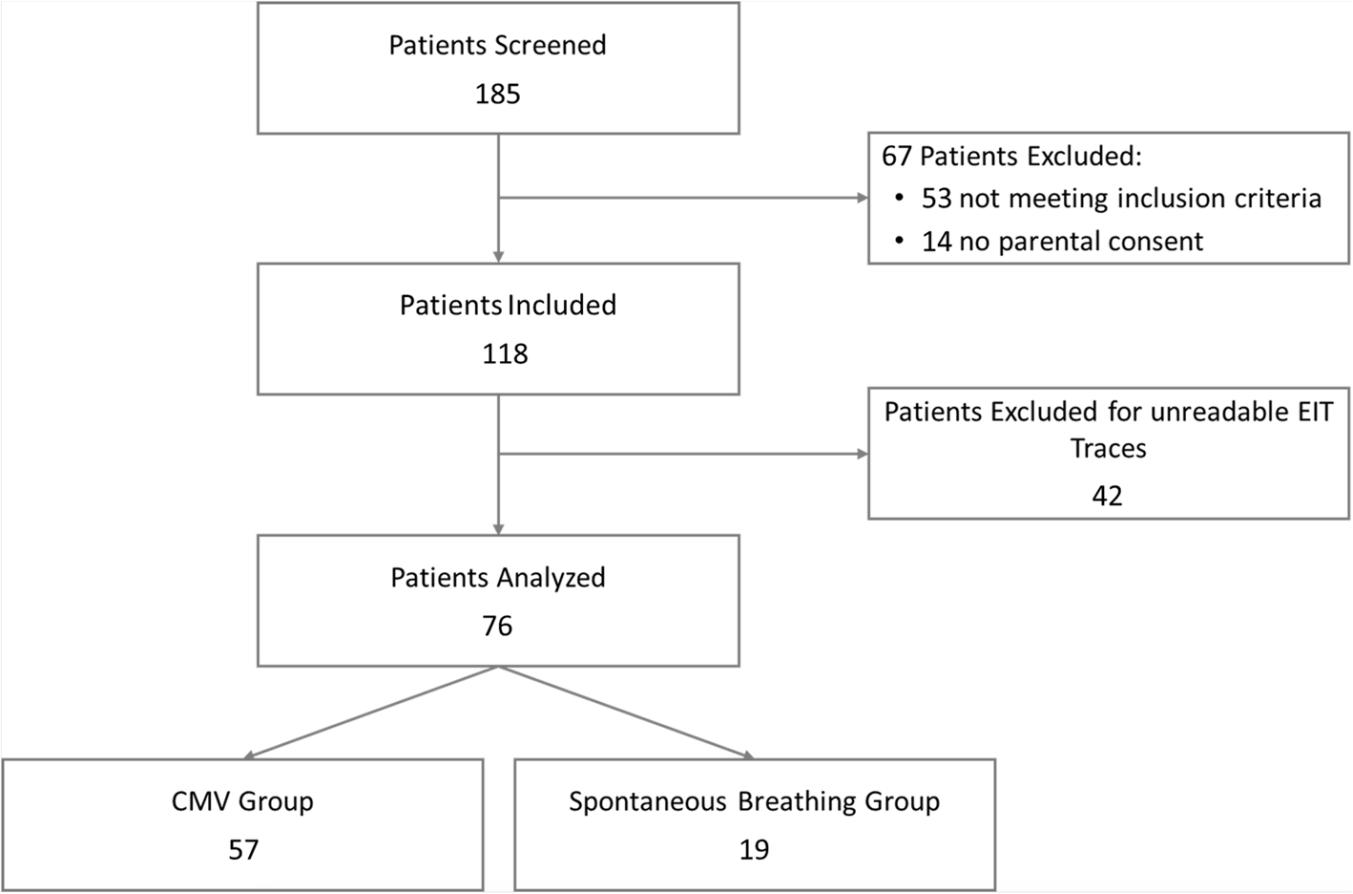
Flow-chart. The process of screening, enrolment, and final analysis is reported. EIT, electrical impedance tomography

From April 2024 to January 2025, 185 patients were screened, 118 were enrolled, and 76 were finally analysed, including 57 in the CMV group and 19 in the spontaneous breathing group (Fig. 1). Table 1 presents the demographic characteristics and surgical procedures of the study population.

**Table 1:** Baseline characteristics

Variables	Missing data	All	CMV	Spontaneous breath
No	–	76	57	19
Age (years)	–	6 (4–8)	6 (4–8)	5 (2–8)
Gender (male)	–	55 (72.4)	43 (75.4)	12 (63.2)
Actual body weight (kg)	1	22 (17–30)	23 (18–30)	18 (13–36)
Height (cm)	1	115 (99–130)	118 (100–130)	107 (90–140)
BMI (kg/m^2^)	1	17 (16–20)	17 (16–20)	16 (15–19)
ASA score	–	1 (1–1)	1 (1–1)	1 (1–1)
Procedure (No./%)				
Urologic surgery	–	16 (21.1)	10 (17.5)	6 (31.6)
ENT surgery	–	28 (36.8)	28 (49.1)	0
Ortho-Trauma surgery	–	14 (18.4)	11 (19.3)	3 (15.8)
Abdominal surgery	–	15 (19.7)	8 (14)	7 (36.8)
Ophthalmic surgery	–	2 (2.6)	0	2 (10.5)
Oral surgery	–	1 (1.3)	1 (1.8)	0

Median and 25–75th percentile (in brackets) or number and percentage (in brackets) are reported. *CMV* controlled mode of mechanical ventilation, *BMI* body mass index, *ASA* American Society of Anesthesiologists, *ENT* surgery, ear-nose-throat surgery

**Fig. 2:**
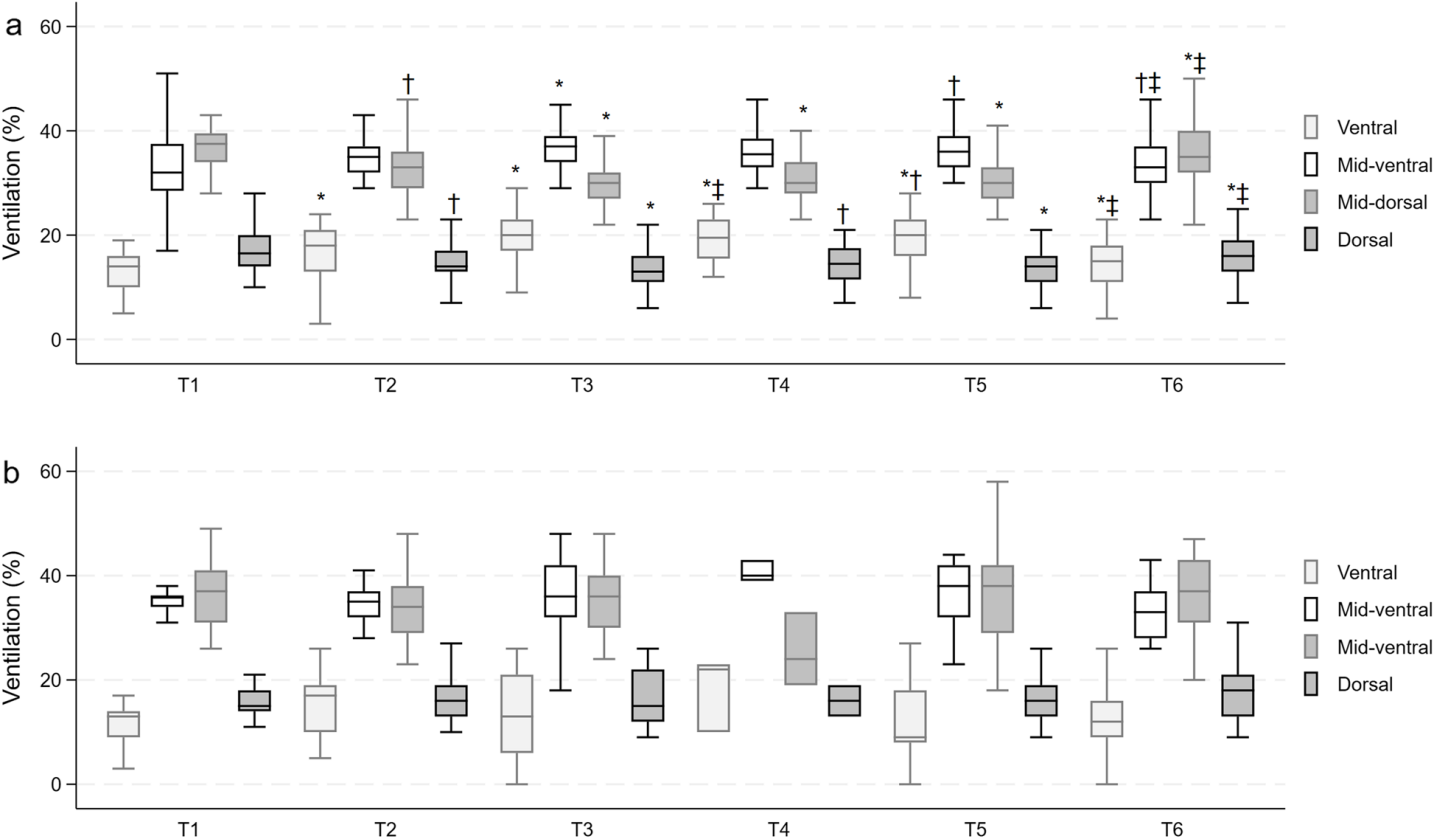
Ventilation distribution. Data of the percentage of ventilation of distribution in ventral, mid-ventral, mid-dorsal, and dorsal region of interest are reported in boxes and whiskers for controlled mode of ventilation (CMV) (**a**) and spontaneous breathing (**b**). *p*-values refer to ANOVA for repeated measures while symbols refer to post hoc test with Bonferroni correction. T1, pre-induction phase with the patient awake and breathing spontaneously; T2, induction phase with assisted ventilation via facial mask; T3, spontaneous breathing or mechanical ventilation at 5 min post-induction; T4, spontaneous breathing or mechanical ventilation at 30 min post-induction; T5, late phase before awakening; T6, end of surgical procedure and/or recovery phase with spontaneous breathing resumed and removal of any airway device used.. Ventral: *p < 0.0001, T2, T3, T4, T5 vs T1; T3 vs T2; T3, T4, T5 vs T6; †*p* < 0.01, T5 vs T2; ‡ *p* < 0.05, T4, T6 vs T2 in CMV. Mid-ventral: *p < 0.0001, T3 vs T1; †*p* < 0.01, T5 vs T1; T6 vs T3; ‡*p* < 0.05, T6 vs T5 in CMV. Mid-dorsal: †*p* < 0.01, T2 vs T1; *p < 0.0001, T3, T4, T5 vs T1; T3 vs T2; T3, T4, T5 vs T6; ‡*p* < 0.05, T6 vs T2 in CMV. Dorsal: †*p* < 0.01, T2, T4 vs T1; *p < 0.0001, T3, T5 vs T1; T3, T5 vs T6; ^‡^*p* < 0.05, T4 vs T6 in CMV

EIT data describing ventilation distribution in CMV and spontaneous breathing groups across the different study time points are depicted in Fig. 2 and Supplementry Table S1.

In the CMV group, the ventral zone exhibited an increase in ventilation from T1 to T2, T3, T4, and T5 (*p* < 0.0001 for all), as well as from T2 to T3 (*p* < 0.0001), T4 (*p* = 0.030), and T5 (*p* = 0.005). In the same region, ventilation was restored to baseline values moving from T2 (*p* = 0.017), T3, T4, and T5 to T6 (*p* < 0.0001 for all). The ventilation increased in the mid-ventral zone from T1 to T3 (*p* < 0.0001) and T5 (*p* = 0.005) while it decreased at T6 compared to T3 (*p* = 0.001) and T5 (*p* = 0.024). A reduction in ventilation was observed in the mid-dorsal region from T1 to T2 (*p* = 0.001), T3, T4, and T5 (*p* < 0.0001 for all) as well as from T2 to T3 (*p* < 0.0001). In the same zone at T6, ventilation was similar to baseline, being increased compared to T2 (*p* = 0.010), T3, T4, and T5 (*p* < 0.0001 for all). In the dorsal area, the distribution of ventilation decreased from baseline to T2 (*p* = 0.005), T3 (*p* < 0.0001), T4 (*p* = 0.001), and T5 (*p* < 0.0001). Conversely, in the same ROI, ventilation distribution returned to baseline values moving from T3 to T6 (*p* < 0.0001), T4 to T6 (*p* = 0.048), and T5 to T6 (*p* < 0.0001). On the contrary, no modifications of ventilation distribution were reported in the group of patients with a preserved spontaneous breath during the entire study duration.

**Fig. 3:**
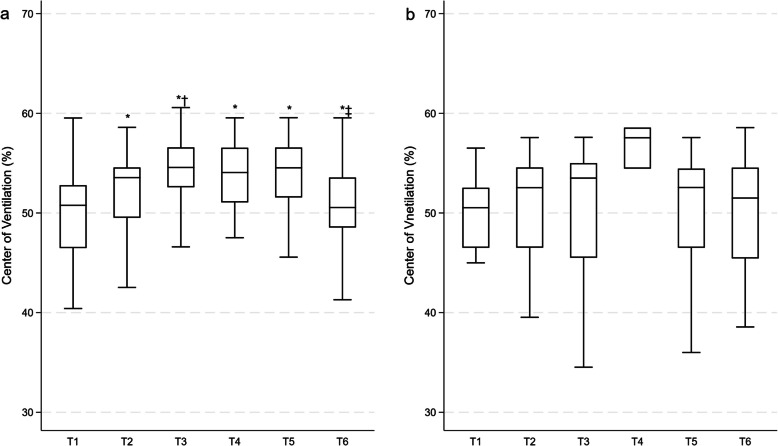
Center of ventilation. The percentage of centre of ventilation is depicted in box and whiskers for controlled ventilation mode (**a**) and spontaneous breathing (**b**). T1, pre-induction phase with the patient awake and breathing spontaneously; T2, induction phase with assisted ventilation via facial mask; T3, spontaneous breathing or mechanical ventilation at 5 min post-induction; T4, spontaneous breathing or mechanical ventilation at 30 min post-induction; T5, late phase before awakening; T6, end of surgical procedure and/or recovery phase with spontaneous breathing resumed and removal of any airway device used. Controlled ventilation mode: *p < 0.0001, T2, T3, T4, T5 vs T1; T3 vs T2; T6 vs T3, T5; †*p* < 0.01: T3 vs T2; ^‡^*p* < 0.05: T6 vs T4 in CMV

In Fig. 3 and Supplementary Table S1, CoV is depicted. Under CMV, CoV progressively shifted towards the ventral zone from T1 to T2, T3, T4, and T5 (*p* < 0.0001 for all) as well as from T2 to T3 (*p* = 0.0001), whereas it returned to baseline values at T6 switching from T3 (*p* < 0.0001, T4 (*p* = 0.002), and T5 (*p* < 0.0001). No significant changes were reported in CoV in patients with preserved spontaneous breathing.

Oxygenation, respiratory system and hemodynamic paremeters are reported in Supplementary information and represented in Table S2 and S3.

In the CMV group, bispectral index, as shown in Table S2, decreased from T1 to all subsequent steps of the study (*p* < 0.0001 for all). Noteworthy, neuromuscular blocking agents were administrated in 42 of 59 (71%) patients in this group at anaesthesia induction. With respect to T2 and T3, bispectral index increased at T5 and T6 (*p* < 0.0001 for all) as well as from T4 to T5 (*p* = 0.002) and T6 (*p* < 0.0001). Finally, at T6, bispectral index was higher than T5 (*p* < 0.0001). Similarly, bispectral index was depressed by sedation employed in the spontaneous breathing group at T2, T3, T4, T5, and T6 compared to baseline (*p* < 0.0001 for all). At T5, bispectral index increased compared to previous time points, namely, T2 (*p* = 0.030) and T3 (*p* = 0.008). Bispectral index continued to rise at T6 when it was greater than values obtained at T2, T3, T4, and T5 (*p* < 0.0001).

**Fig. 4:**
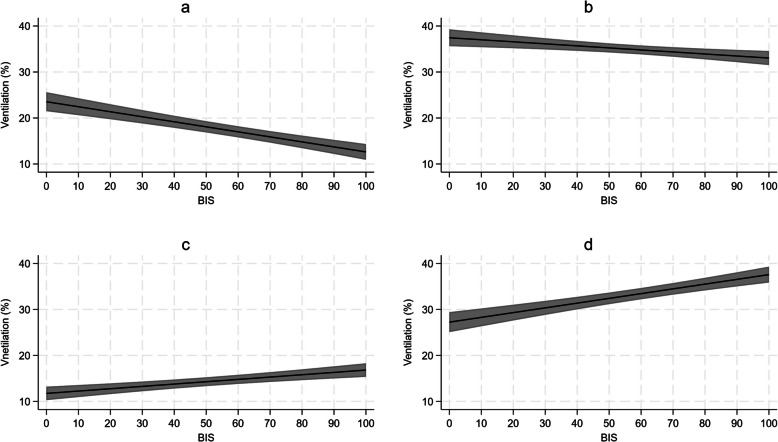
Multilevel mixed-effects linear regression model for the prediction of ventilation distribution according to bispectral index in the patients undergoing controlled mechanical ventilation

Figure 4 represents the MMEM for prediction of ventilation distribution according to bispectral index in the CMV group. The model estimates indicated that the ventilation increased with the progressive reduction of bispectral index in ventral (coefficient – 0.109, standard error 0.015, 95% confidence interval − 0.138 to − 0.080, *p* < 0.0001) and mid-ventral regions (coefficient − 0.044, standard error 0.014, 95% confidence interval − 0.071 to − 0.017, *p* = 0.002). On the contrary, the model estimates suggested a progressive reduction of the ventilation in dorsal (coefficient 0.051, standard error 0.011, 95% confidence interval: 0.028 to 0.073, *p* < 0.0001) and mid-dorsal zone (coefficient 0.103, standard error 0.015, 95% confidence interval 0.073 to 0.133, *p* < 0.0001) in response to bispectral index reduction. No modifications to the reported effects were observed after adjusting the model on patients’ characteristics, namely age and BMI.

**Fig. 5:**
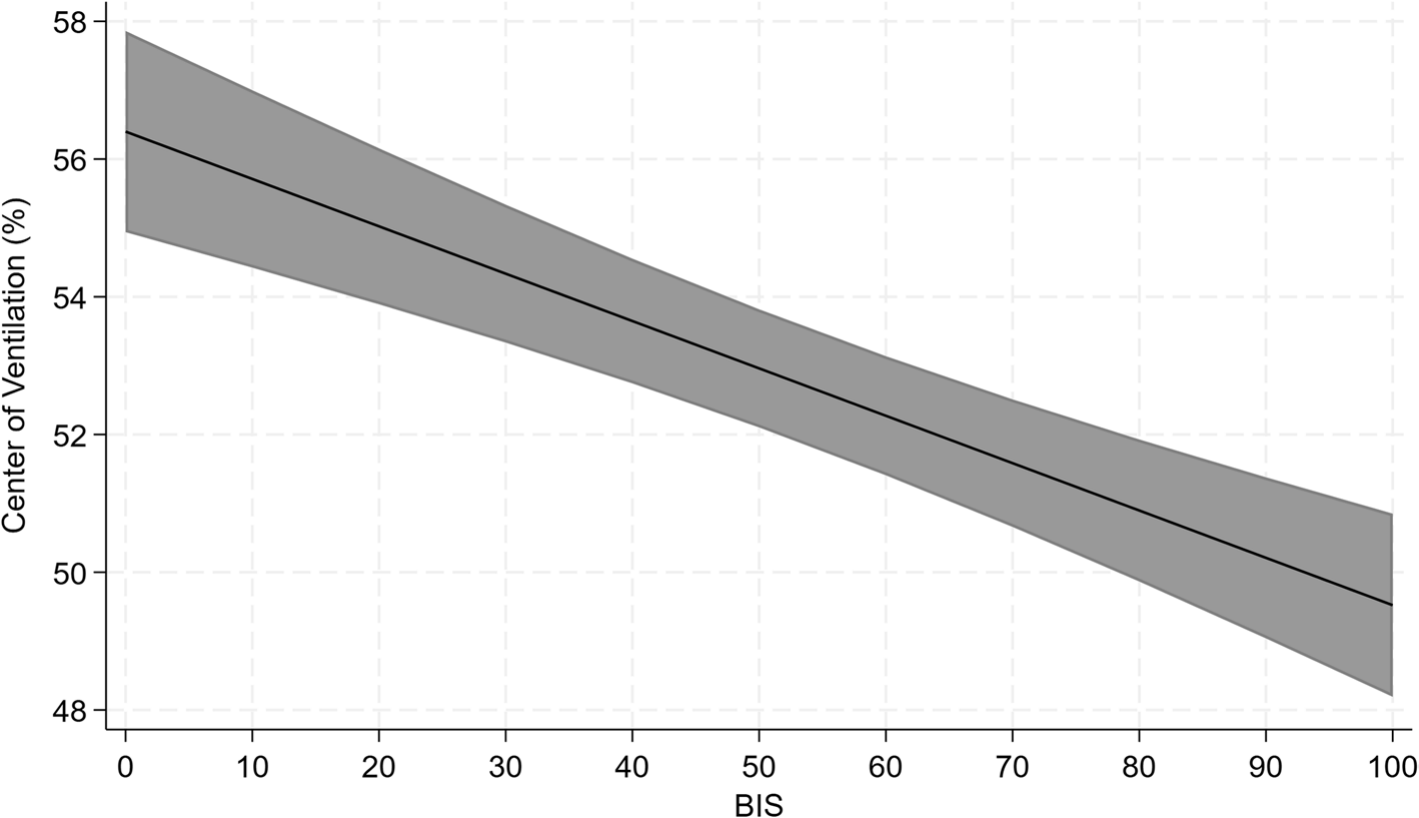
Multilevel mixed-effects linear regression model for the prediction of center of ventilation modifications according to bispectral index in the patients undergoing controlled mechanical ventilation

The MMEM for the prediction of CoV according to bispectral index in the CMV group is represented in Fig. 5. The model estimates identified that, at decreasing of bispectral index, a progressive migration of CoV towards non-dependent lung regions was observed (coefficient − 0.069, standard error 0.011, 95% confidence interval − 0.091 to − 0.047, *p* < 0.0001). Also in this case, no impacts to the reported effects were observed by adjusting the model on patients’ characteristics, namely age and BMI.

In the spontaneous breathing group, no significant relationship was obtained for the prediction of ventilation distribution and CoV migration based on bispectral index.

## Discussion

The present study provides an in-depth analysis of lung ventilation distribution patterns following CMV application or spontaneous breathing preservation in paediatric patients undergoing surgery.

The main findings of the present investigation can be summarised as follows: (1) in contrast to spontaneous breathing conditions, where ventilation distribution was preserved throughout the entire study duration, CMV led to a progressive shift in ventilation towards ventral lung ROIs at the expense of dorsal lung zones, (2) under CMV, the ventilation redistribution observed during anaesthesia reverted by the end of the procedure, coinciding with the restoration of spontaneous breathing and the return of lung aeration to baseline conditions; and (3) in patients subjected to CMV, deeper anaesthesia levels, as indicated by the BIS, more or less associated to mioresolution, were associated with a predominant redistribution of ventilation towards non-dependent lung ROIs rather than dependent ones.

General anaesthesia and CMV are required in many medical and surgical procedures in paediatric populations [14]. However, their effects on respiratory function remain an area of active research. To address this gap, we evaluated the intraoperative application of EIT-driven advanced respiratory monitoring, which effectively allows a dynamic and real-time assessment of ventilation modifications occurring during surgery [7].

The ventilation pattern observed under CMV using EIT was consistent with previous findings showing that ventilation in posterior pulmonary zones decreased from 54 to 49% with the same ventilatory mode, in favour of a concurrent increased distribution to non-dependent lung ROIs [13]. This shift has been attributed to a reduction in functional residual capacity (FRC) and small airway occlusion [15]. The supine position further exacerbates this phenomenon by promoting gravitational compression of dorsal lung regions, increasing pulmonary atelectasis and impairing gas exchange [13]. This effect is particularly relevant in paediatric patients, due to their unique anatomical and physiological characteristics, resulting in higher chest wall compliance and lower FRC compared to adults, which predispose them to derecruitment under CMV [12].

In line with previous findings, in the CMV group, ventilation distribution returned to pre-induction conditions at the end of anaesthesia, suggesting that these changes are reversible upon the recovery of spontaneous breathing [13]. This phenomenon highlights the transient nature of CMV-induced alterations, with a subsequent restoration of normal respiratory physiology at the end of anaesthesia. The recovery of baseline respiratory patterns suggests that, although significant, anaesthesia-induced alterations may not lead to long-term consequences in patients with healthy lung function. However, it would be useful to investigate whether this recovery is less efficient in patients with pre-existing respiratory conditions, emphasizing the importance of continuous respiratory monitoring during and immediately after surgery. In these circumstances, it is possible that EIT could be a valuable tool to guide and tailor intraoperative ventilation settings. Personalised ventilatory strategies, such as moderate positive end-expiratory pressure and recruitment manoeuvres, might mitigate ventilation redistribution, maintaining alveolar recruitment and improving oxygenation, as seen in the adult population undergoing general surgery [16]. Advanced respiratory monitoring via EIT could therefore offer significant benefits in managing ventilation, particularly in surgical patients at risk of postoperative pulmonary complications, with the aim of mitigating the impact of CMV, general anaesthesia, and surgery on postoperative pulmonary function.

In our setting, patients who preserved spontaneous breathing exhibited stable ventilation distribution across all study phases. This observation contrasts with previous findings obtained in spontaneously breathing paediatric patients (aged 1–6 years) [12], where deep sedation for magnetic resonance imaging procedures led to a redistribution of ventilation with an increased predominance of dependent atelectasis. This discrepancy is likely attributable to the lighter sedation plan adopted in our study compared to the previous setting, which may have influenced the respiratory mechanics [12].

The observed link between deeper levels of anaesthesia, associated with neuromuscular block in 71% of the cases, and a shift in ventilation distribution towards ventral ROIs in the CMV group underscores the critical role played by anaesthesia depth and mioresolution in the complex modulation of the respiratory system mechanics during surgery. As BIS decreases, indicating deeper anaesthesia, and neuromuscular block is assured, diaphragmatic relaxation becomes more pronounced, leading to a cephalad shift of its dorsal portion resulting in an increased transmural pressure and a consequent redistribution of pulmonary ventilation from the dorsal regions to non-dependent lung zones [12, 13]. This redistribution may exacerbate ventilation-perfusion mismatch, potentially compromising oxygenation in vulnerable patients.

This study has several limitations that require disclosure. It is an observational study designed to describe the intraoperative application of EIT to assess the distribution of lung ventilation. The study did not specifically assess the distribution of intraoperative ventilation in predefined subgroups of the paediatric population stratified by age or BMI, and no significant impact of these factors was observed in our cohort. This finding is likely attributable to the fact that the age and BMI of the study population were predominantly in the range of 4–8 years and 16–20 kg/m^2^, respectively. Therefore, future research should address and overcome this limitation by assessing ventilation distribution during surgery in selected paediatric subgroups. Also, the study focused on the paediatric age range up to 12 years, as this was the most represented group in our centre. Therefore, our findings may not be directly applicable to adolescents aged 14–17 years. Due to the monocentric nature of this study, the generalisability of the findings is limited. Future studies should explore the impact of different ventilation strategies on the observed ventilation redistribution and evaluate their long-term outcomes.

## Conclusion

CMV and deep anaesthesia, more or less combined with neuromuscular block, were associated with progressive changes in lung aeration, leading to a gradual shift of ventilation towards the ventral lung regions at the expense of the dorsal ROIs in paediatric patients undergoing surgery. A better understanding of these changes may help in tailoring mechanical ventilation strategies to optimise intraoperative respiratory mechanics and potentially reduce postoperative complications. Further research is needed to confirm these findings and to explore the factors influencing intraoperative respiratory mechanical properties across different surgical specialties.

## Supplementary Information

Supplementary Material 1: Table S1. Distribution of ventilation. Table S2. Oxygenation and respiratory system. Table S3. Hemodynamics and depth of anesthesia.

## Data Availability

No datasets were generated or analysed during the current study.

## Abbreviations

ASA: American Society of Anesthesiologists
BIS: Bispectral index
BMI: Body mass index
CMV: Controlled mechanical ventilation
CoV: Center of ventilation
EIT: Electrical impedance tomography
ENT: Ear-nose-throat
FRC: Functional residual capacity
MMEM: Multilevel mixed-effects linear regression model
ROI: Region of interest
