# Supplementary material for: Patterns of lung aeration assessed through electrical impedance tomography in paediatric patients undergoing elective surgery: insights from a prospective and observational data-registry
Source: J Anesth Analg Crit Care. 2025 Jun 23;5:34. doi: 10.1186/s44158-025-00254-x (PMC12183812; doi:10.1186/s44158-025-00254-x)
Supplement: Supplementary file 1 — Supplementary Material 1: Table S1. Distribution of ventilation. Table S2. Oxygenation and respiratory system. Table S3. Hemodynamics and depth of anesthesia. [file 44158_2025_254_MOESM1_ESM.docx]

**Oxygenation, respiratory system and hemodynamics parameters**

Peripheral oxygen saturation did not change across the different study time points in CMV group. In contrast, in spontaneous breathing group, it decreased at T6 compared to T2 and T3 (p= 0.001 for both). A lower inspiratory oxygen fraction was set at T3, T4, T5, and T6 compared to T2 (p<0.0001 for all) in patients undergoing CMV. In the spontaneous breathing group, the inspiratory oxygen fraction was reduced solely at T6 compared to the previous steps (T6 vs T2, T3 and T5, p<0.0001; T6 vs T4, p= 0.008). In CMV group, respiratory rate varied at T2, T3, T4, T5, and T6 compared to baseline (T2 vs T1, p= 0.002; T3, T4 vs T1, p< 0.0001; T5 vs T1 p=0.001; T6 vs T1 p=0.004) and decreased at T6 compared to previous time points encompassing T2, T3, T4, and T5 (p<0.0001 for all). On the contrary, no changes in respiratory rate were reported in spontaneous breathing group during whole study duration. In CMV, tidal volume diminished at T4 compared to T3 (p=0.032). Tidal volume reduced at T4 compared to T3 in CMV (p= 0.032) whereas PEEP, mean airway pressure, and plateau airway pressure, inspiratory showed no significant modifications during the entire study duration.

Hemodynamic parameters and depth of anaesthesia are described in table 3. Contrariwise to spontaneous breathing group, where no modifications were observed, in CMV group an increase in heart rate at T6 compared to the previous time-points of the study (p<0.0001 for all) was reported. In the CMV group, non-invasive systolic blood pressure decreased from baseline to T2, T3, T4 (p<0.0001 for all), and T5 (p=0.012) and increased at T6 compared to T2, T3, and T4 (p<0.0001 for all). In the spontaneous breathing group, there was a reduction in non-invasive systolic blood pressure at T3 (p= 0.001), T5 (p<0.0001), and T6 (p= 0.007), compared to T1. In CMV group, non-invasive diastolic blood pressure decreased from T1 to T2 (p=0.002), T3 (p<0.0001), and T4 (p=0.002) and increased at T6 with respect to T2 (p= 0.037) and T3 (p< 0.0001). Non-invasive diastolic blood pressure was diminished at T3 (p=0.002) T5 (p=0.046) and T6 (p=0.019) compared to baseline as well as T3 compared to T2 (p=0.033).

**Table S1.** Distribution of ventilation

| Parameters | T1 | T2 | T3 | T4 | T5 | T6 | *p*-value |
| --- | --- | --- | --- | --- | --- | --- | --- |
| Ventral ROI (%) | | | | | | | |
| *CMV* | 14 (10-16) [40] | 18 (13-21) [57]* | 20 (17-23) [57]* | 19.5 (15.5-23) [32]*^‡^ | 20 (16-23) [55]*^†^ | 15 (11-18) [57]*^‡^ | <0.0001 |
| *Spontaneous breath* | 13 (9-14) [13] | 17 (10-19) [19] | 13 (6-21) [19] | 22 (10-23) [3] | 9 (8-18) [13] | 12 (9-16) [19] | 0.053 |
| Mid-ventral ROI (%) | | | | | | | |
| *CMV* | 32 (28.5-37.5) [40] | 35 (32-37) [57] | 37 (34-39) [57]* | 35.5 (33-38.5) [32] | 36 (33-39) [55]^†^ | 33 (30-37.5) [57]^†‡^ | <0.0001 |
| *Spontaneous breath* | 36 (34-36) [13] | 35 (32-37) [19] | 36 (32-42) [19] | 40 (39-43) [3] | 38 (32-43) [13] | 33 (28-37) [19] | 0.756 |
| Mid-dorsal ROI (%) | | | | | | | |
| *CMV* | 37.5 (34-39.5) [40] | 33 (29-36) [57]^†^ | 30 (27-32) [57]* | 30 (28-34) [32]* | 30 (27-33) [55]* | 35 (32-40) [57]*^‡^ | <0.0001 |
| *Spontaneous breath* | 37 (31-41) [13] | 34 (29-38) [19] | 36 (30-40) [19] | 24 (19-33) [3] | 38 (29-42) [13] | 37 (31-43) [19] | 0.175 |
| Dorsal ROI (%) | | | | | | | |
| *CMV* | 16.5 (14-20) [40] | 14 (13-17) [57]^†^ | 13 (11-16) [57]* | 14.5 (11.5-16.5) [32]^†^ | 14 (11-16) [55]* | 16 (13-19) [57]*^‡^ | <0.0001 |
| *Spontaneous breath* | 15 (14-18) [13] | 16 (13-19) [19] | 15 (12-22) [19] | 16 (13-19) [3] | 16 (13-19) [13] | 18 (13-21) [19] | 0.405 |
| Center of Ventilation (%) | | | | | | | |
| *CMV* | 50.8 (46.5-52.8) [40] | 53.6 (49.5-54.6) [57]* | 54.6 (52.6-56.6) [57]*^†^ | 54.1 (51.1-56.6) [32]* | 54.5 (51.6-56.6) [55]* | 50.6 (48.5-53.6) [57]^‡^* | <0.0001 |
| *Spontaneous breath* | 50.5 (46.5-52.5) [13] | 52.5 (46.5-54.6) [19] | 53.5 (45.5-55) [19] | 57.6 (54.5-58.6) [3] | 52.6 (46.5-54.5) [13] | 51.5 (45.4-54.6) [19] | 0.594 |

Data are presented in median and 25^th^-75^th^ percentile. *P*-values refer to ANOVA for repeated measures while symbols refer to post hoc test with Bonferroni correction. T1, pre-induction phase with the patient awake and breathing spontaneously; T2, induction phase with assisted ventilation via facial mask; T3 spontaneous breathing or mechanical ventilation at 5 minutes post-induction; T4, spontaneous breathing or mechanical ventilation at 30 minutes post-induction; T5, late phase before awakening; T6, end of surgical procedure and/or recovery phase with spontaneous breathing resumed and removal of any airway device used; region of interest (ROI); controlled mode of ventilation (CMV).

Ventral ROI: * p<0.0001, T2, T3, T4, T5 vs T1; T3 vs T2; T3, T4, T5 vs T6; † p< 0.01, T5 vs T2; ‡ p<0.05: T4, T6 vs T2 in CMV.

Mid-ventral ROI: * p<0.0001, T3 vs T1; † p< 0.01: T5 vs T1; T6 vs T3; ‡ p<0.05: T6 vs T5 in CMV

Mid-dorsal ROI: † p< 0.01, T2 vs T1; * p<0.0001, T3, T4, T5 vs T1; T3 vs T2; T3, T4, T5 vs T6; ‡ p<0.05: T6 vs T2 in CMV

Dorsal ROI: † p< 0.01: T2, T4 vs T1; * p<0.0001, T3, T5 vs T1; T3, T5 vs T6; ^‡^ p<0.05: T4 vs T6. In CMV

Center of Ventilation: * p<0.0001, T2, T3, T4, T5 vs T1; T3 vs T2; T6 vs T3, T5; † p< 0.01, T3 vs T2; ^‡^ p<0.05, T6 vs T4 in CMV

**Table S2**. Oxygenation and respiratory system

| **Parameters** | **T1** | **T2** | **T3** | **T4** | **T5** | **T6** | ***p*-value** |
| --- | --- | --- | --- | --- | --- | --- | --- |
| **Peripheral oxygen saturation (%)** | | | | | | | |
| *CMV* | 100 (99-100) [36] | 100 (100-100) [56] | 100 (100-100) [55] | 100 (100-100) [37] | 100 (100-100) [48] | 98 (99-100) [55] | 0.549 |
| *Spontaneous breath* | 100 (99-100) [8] | 100 (100-100) [19] | 100 (100-100) [19] | 100 (100-100) [5] | 100 (99-100) [10] | 99 (98-100) [18]^†^ | 0.0006 |
| **Inspiratory oxygen fraction** | | | | | | | |
| *CMV* | - | 1 (1-1) [56] | 0.4 (0.4-0.4) [55]* | 0.4 (0.4-0.4) [37]* | 0.4 (0.4-0.4) [48]* | 0.21 (0.21-1) [55]* | <0.0001 |
| *Spontaneous breath* | - | 1 (1-1) [19] | 1 (1-1) [18] | 1 (1-1) [5] | 1 (1-1) [10] | 0.21 (0.21-1) [18]*^†^ | <0.0001 |
| **Respiratory rate (breaths*min^-1^)** | | | | | | | |
| *CMV* | 20 (17-25) [44] | 24 (21-25) [50]^†^ | 25 (22-27) [56]* | 25 (23-27) [37]* | 25 (22-27) [49]^†^ | 16 (15-20) [44]^†^* | <0.0001 |
| *Spontaneous breath* | 20 (15-25) [11] | 21 (16-25) [18] | 20 (16-25) [16] | 22 (20-23) [4] | 20 (18-22) [10] | 20 (16-22) [16] | 0.889 |
| **Tidal volume (ml)** | | | | | | | |
| *CMV* | - | - | 180 (140-210) [56] | 170 (140-210) [37]^‡^ | 165 (140-210) [48] | - | 0.035 |
| *Spontaneous breath* | - | - | - | - | - | - |  |
| **Positive end-expiratory pressure (cmH_2_O)** | | | | | | | |
| *CMV* | - | - | 5 (5-5) [56] | 5 (5-5) [37] | 5 (5-5) [49] | - | 0.999 |
| *Spontaneous breath* | - | - | - | - | - | - |  |
| **Mean airway pressure (cmH_2_O)** | | | | | | | |
| *CMV* | - | - | 10 (9-11) [51] | 10 (9-11) [34] | 10 (9-11) [45]^‡^ | - | 0.097 |
| *Spontaneous breath* | - | - | - | - | - | - |  |
| **Plateau airway pressure (cmH_2_O)** | | | | | | | |
| *CMV* | - | - | 15 (13-16) [40] | 14 (13-16) [27] | 15 (14-18) [36] | - | 0.900 |
| *Spontaneous breath* | - | - | - | - | - | - |  |

Data are presented in median and 25^th^-75^th^ percentile in round brackets along with number of observations in square brackets. CMV, controlled mode of ventilation. P-values refer to repeated measures ANOVA while symbols refer to post hoc test with Bonferroni’s correction. T1, pre-induction phase with the patient awake and breathing spontaneously; T2, induction phase with assisted ventilation via facial mask; T3 spontaneous breathing or mechanical ventilation at 5 minutes post-induction; T4, spontaneous breathing or mechanical ventilation at 30 minutes post-induction; T5, late phase before awakening; T6, end of surgical procedure and/or recovery phase with spontaneous breathing resumed and removal of any airway device used.

Peripheral oxygen saturation: † p< 0.01: T6 vs T2, T3 in spontaneous breath.

Inspired oxygen fraction: * p<0.0001, T2 vs T3, T4, T5, T6 in CMV; * p<0.0001, T2, T3, T5 vs T6; † p< 0.01: T4 vs T6 in spontaneous breath.

Respiratory rate: † p< 0.01 T2, T5, T6 vs T1; * p<0.0001 T2, T3, T4, T5 vs T6 in CMV.

Tidal volume: ‡ p< 0.05: T4 vs T3 in CMV.

Table S3. Hemodynamics and depth of anesthesia

| **Parameters** | **T1** | **T2** | **T3** | **T4** | **T5** | **T6** | ***p*-value** |
| --- | --- | --- | --- | --- | --- | --- | --- |
| **Heart rate (beats*min^-1^)** | | | | | | | |
| *CMV* | 93 (78-100) [35] | 85 (76-98) [56] | 91 (80-103) [55] | 94 (82-101) [37] | 88 (78-101) [48] | 101 (93-120) [55]* | <0.0001 |
| *Spontaneous breath* | 96 (78-115) [8] | 83 (79-114) [19] | 91 (86-106) [19] | 97 (87-99) [5] | 97 (91-110) [10] | 89 (84-100) [18] | 0.243 |
| **Non-invasive systolic blood pressure (mmHg)** | | | | | | | |
| *CMV* | 112 (104-118) [31] | 100 (92-110) [54]* | 96 (89-107) [54]* | 99 (87-114) [36]* | 100 (90-115) [47]^‡^ | 110 (100-120) [52]^†^* | <0.0001 |
| *Spontaneous breath* | 110 (103-117) [8] | 99 (95-108) [17] | 95 (89-99) [17]^†^ | 95 (94-99) [5] | 91 (84-99) [10]* | 95 (90-102) [17]^†^ | 0.0001 |
| **Non-invasive diastolic blood pressure (mmHg)** | | | | | | | |
| *CMV* | 64 (57-79) [31] | 55 (50-60) [54]^†^ | 50 (42-58) [54]* | 51 (43-63) [36]^†^ | 60 (47-70) [47] | 60 (53-71) [51]^‡^* | <0.0001 |
| *Spontaneous breath* | 70 (63-75) [8] | 58 (54-67) [17]^†^ | 49 (43-54) [17]^‡^ | 50 (45-59) [5] | 57 (40-64) [10]^‡^ | 55 (45-64) [17]^‡^ | 0.0008 |
| **Bi-spectral index** | | | | | | | |
| *CMV* | 95 (90-97) [25] | 40 (35-51) [40]* | 45 (38-54) [43]* | 46 (42-58) [31]* | 58 (49-65) [41]*^†^ | 78 (75-83) [42]* | <0.0001 |
| *Spontaneous breath* | 94 (88-96) [11] | 44 (40-46) [15]* | 40 (38-48) [15]* | 48 (43-51) [4]* | 58 (52-64) [10]*^‡†^ | 74 (66-82) [15]* | <0.0001 |

Data are presented in median and 25^th^-75^th^ percentile in round brackets along with number of observations in square brackets. CMV, controlled mode of ventilation. P-values refer to repeated measures ANOVA while symbols refer to post hoc test with Bonferroni’s correction. T1, pre-induction phase with the patient awake and breathing spontaneously; T2, induction phase with assisted ventilation via facial mask; T3 spontaneous breathing or mechanical ventilation at 5 minutes post-induction; T4, spontaneous breathing or mechanical ventilation at 30 minutes post-induction; T5, late phase before awakening; T6, end of surgical procedure and/or recovery phase with spontaneous breathing resumed and removal of any airway device used.

Heart rate: *p <0.0001: T6 vs T1, T2, T3, T4, T5 in CMV.

Non-invasive systolic blood pressure: *p <0.0001 T2, T3, T4, vs T1; T6 vs T3; T6 vs T2, T3, T4; ‡ p <0.05: T5 vs T1; in CMV. *p <0.0001 T5 vs T1; † p<0.01: T3, T6 vs T1 in spontaneous breathing.

Non-invasive diastolic blood pressure: *p <0.0001, T3 vs T1; T6 vs T3; † p<0.01: T2, T4 vs T1; ‡ p <0.05: T6 vs T2 in CMV; † p<0.01: T3 vs T1; ‡ p <0.05 T5, T6 vs T1; T3 vs T2 in spontaneous breathing.

Bispectral index: * p <0.0001 T2, T3, T4, T5, vs T1; T5 vs T2, T3; T6 vs T2, T3, T4, T5; † p< 0.01: T5 vs T4 in CMV; *p <0.0001 T2, T3, T4, T5, T6 vs T1; T6 vs T2, T3, T4, T5; ‡ p <0.05 T5, vs T2; † p< 0.01: T5 vs T3 in spontaneous breath
